# A Wide Complex Tachycardia in a Structurally Normal Heart: What is the Mechanism? Where to Ablate?

**DOI:** 10.19102/icrm.2019.100101

**Published:** 2019-01-15

**Authors:** Yash Lokhandwala, Aniruddha Vyas

**Affiliations:** ^1^Cardiology Department, Holy Family Hospital and Research Center, Mumbai, India; ^2^Cardiology Department, Medanta Hospital, Indore, India

**Keywords:** Antedromic tachycardia, distal ablation in atriofascicular Mahaim pathway, Mahaim pathway, pre-excited atrial fibrillation via Mahaim pathway

## Abstract

We present a case of wide complex tachycardia (left bundle branch block, superior axis) in a 30-year-old female with a structurally normal heart and no baseline preexcitation on electrocardiogram. Tachycardia initiation and atrial pacing confirmed the existence and participation of a right-sided atriofascicular decrementally conducting Mahaim pathway. Tachycardia mechanism validation by atrial pacing maneuvers was documented as well as was a response to adenosine and preexcited Mahaim atrial fibrillation. The usual ablation site did not record a Mahaim potential; hence, ablation was performed at a site other than the usual one.

## Case presentation

A 30-year-old female with normal baseline electrocardiogram (ECG) and recurrent drug-refractory wide QRS tachycardia was evaluated using electrophysiology study (EPS). All documented tachycardia episodes (rate: approximately 180 bpm) showed a QRS morphology of left bundle branch block (LBBB) with left axis on the ECG. During EPS, evidence of decremental conduction via a Mahaim (atriofascicular) pathway was documented along with progressive widening of the QRS (LBBB, left axis) during right atrial pacing at successively shorter cycle lengths. This was associated with increasing A–H intervals and reversal of septal activation sequence as the preexcitation over Mahaim overtook atrioventricular (AV) nodal conduction. Antidromic tachycardia with LBBB, left axis, using this Mahaim pathway was induced. We also documented preexcited (anterograde Mahaim conduction) atrial fibrillation (AF), a finding that is less commonly seen in these cases.1 During mapping, no Mahaim potential was recorded during tachycardia/right atrial pacing along the tricuspid annulus.

## Discussion

**Figure 1:**
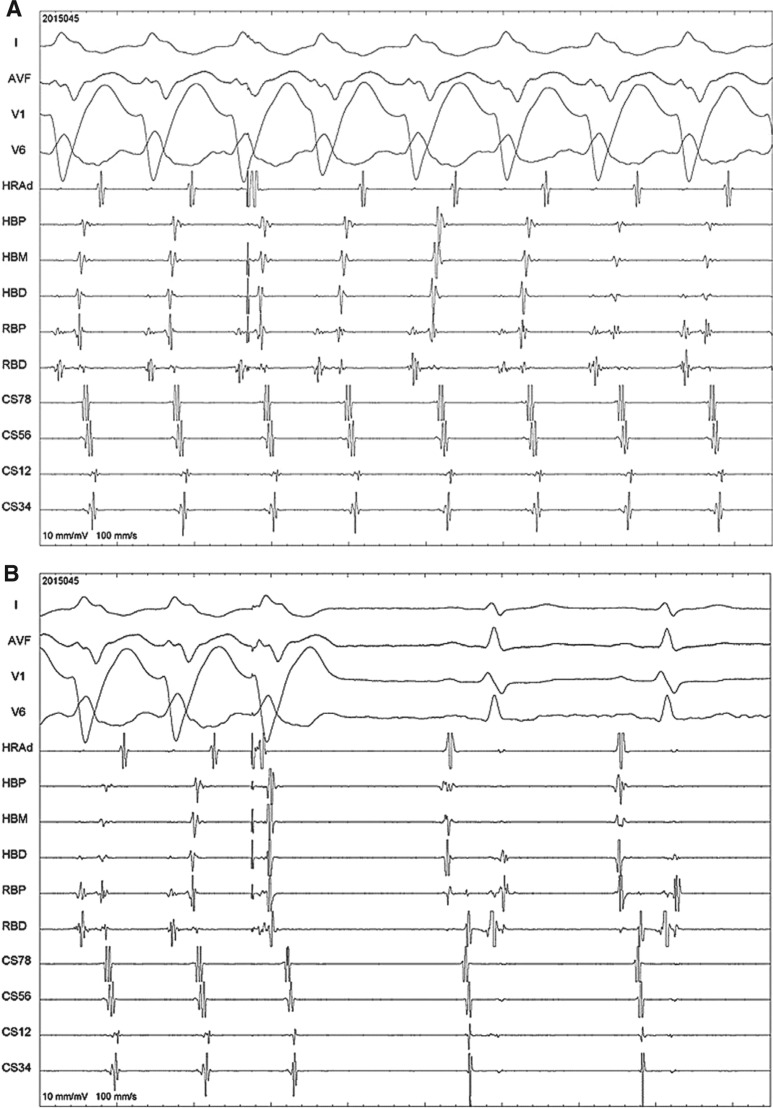
**A and B:** APD delivered from the right atrial free wall during tachycardia. Shown here are surface ECG leads I, aVF, V1, and V6 as well as intracardiac recordings. HRA: high right atrium; HBP/M/D: His bundle proximal, mid, and distal; RBP/D: right bundle proximal and distal; CS: coronary sinus (higher numbers mean proximal electrodes and vice versa).

**Figure 1A** shows delayed right free-wall atrial premature delivery (APD) after the septal A was committed and preexcited the V, indicating the presence of an accessory pathway (AP); it also resets the tachycardia, clearly indicating the participation of the AP in the tachycardia circuit. At times, right free-wall APD can postexcite the ventricle/tachycardia, which confirms the presence and participation of the AP as the anterograde limb of tachycardia.

**Figure 1B** shows that the tachycardia is terminated with an APD without conducting to the ventricle, indicating that the atrium is a component of the circuit. Rarely, termination without conducting to the ventricles can also be seen in AV nodal reentrant tachycardia (AVNRT) as well. An APD from a site close to the AP (the right free wall in this case) terminating the tachycardia without conducting to the ventricle does not rule out the possibility of AVNRT with a bystander Mahaim conduction, unless this occurs without advancement of septal atrial activation (coronary sinuses 7 and 8 in this case). Here, the antidromic wavefront from the premature atrial complex collides with the tachycardia wavefront above the septal atrial activation. Hence, termination is in the anterograde limb only (namely, the AP) when the septal atrium is refractory. This finding conclusively demonstrates that the AP participates in the tachycardia circuit and is not just a bystander with AVNRT.

**Figure 2:**
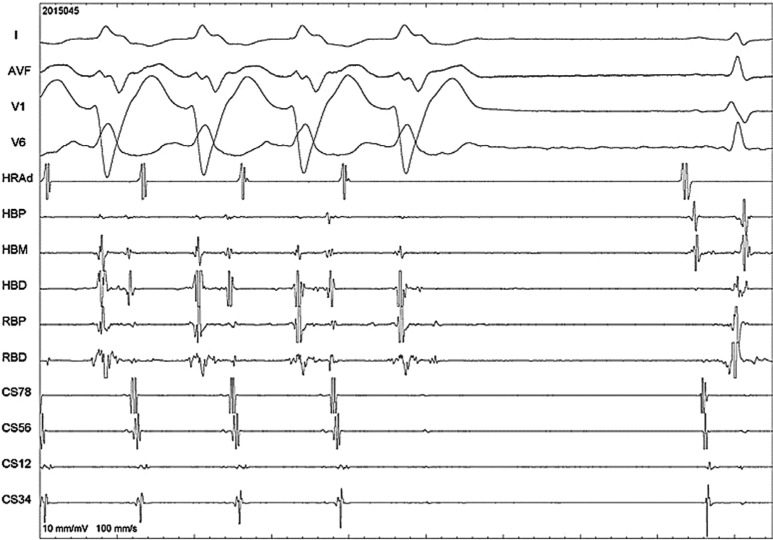
Response to 6-mg adenosine intravenous injection. Shown here are surface ECG leads I, aVF, V1, and V6 as well as intracardiac recordings. HRA: high right atrium; HBP/M/D: His bundle proximal, mid, and distal; RBP/D: right bundle proximal and distal; CS: coronary sinus (higher numbers mean proximal electrodes and vice versa).

In **Figure 2**, the tachycardia is terminated in the AV node as a response to intravenous adenosine administration.

**Figure 3:**
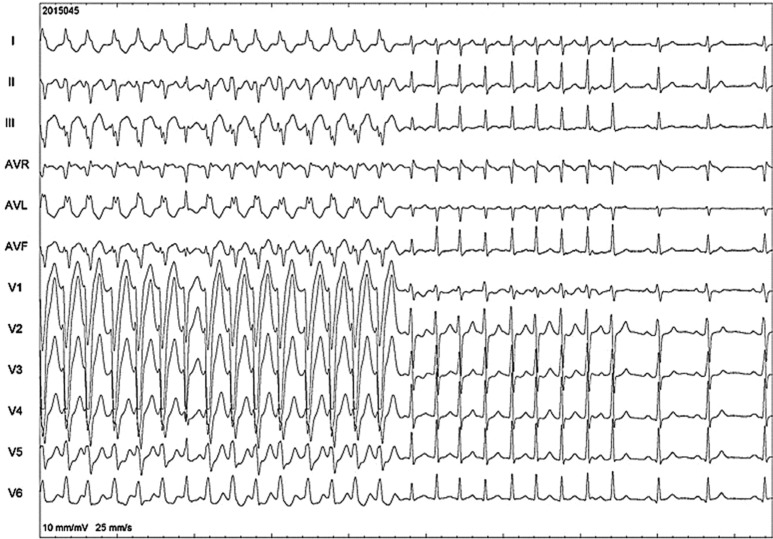
A 12-lead ECG documented this tachycardia during the study (see Discussion section).

In the left half of **Figure 3**, we document a preexcited AF (which is a very uncommon phenomenon with Mahaim pathway). In the right half of **Figure 3**, we see a sudden loss of preexcitation as the AP gets bumped during catheter positioning. AF spontaneously terminated to the sinus rhythm, as seen at the right end of the image.

**Figure 4:**
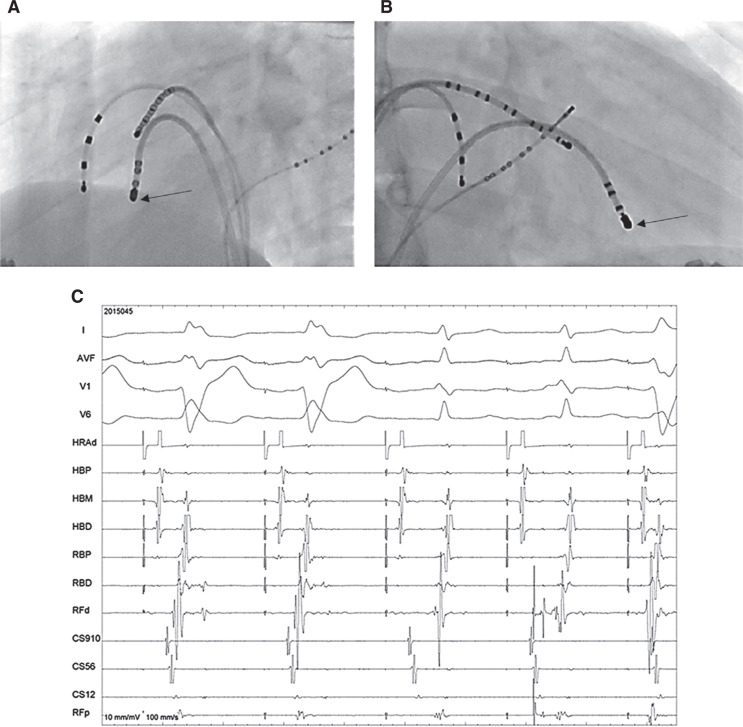
Ablation site (black arrow) in the left **(A)** and right anterior oblique **(B)** images. **C:** Corresponding electrograms at the successful ablation site during radiofrequency energy delivery. Shown here are surface ECG leads I, aVF, V1, and V6 as well as intracardiac recordings. HRA: high right atrium; HBP/M/D: His bundle proximal, mid, and distal; RBP/D: right bundle proximal and distal; CS: coronary sinus (higher numbers mean proximal electrodes and vice versa); RFd/p: ablation catheter distal and proximal electrodes.

In **Figure 4**, often, a usual mapping protocol for ablation would involve mapping the lateral tricuspid annulus for Mahaim potential, but we could not record it in this case. Instead, distal insertion sites near the right bundle can be mapped during right atrial pacing (to bring out the preexcitation) for earliest local V for the delivery of ablation energy as a reasonable alternative in circumstances when Mahaim potential cannot be recorded along the tricuspid annulus.2,3 The mapping for earliest local V can also be performed during tachycardia, but it may compromise the stability of the catheter to operate during ablation with abrupt termination of tachycardia. The fluoroscopy frames in the left and right anterior oblique views show the successful ablation site recording the signals as depicted in the lower frame with disappearance of preexcitation during ablation. The last beat seen in the frame with a wider QRS and a morphology similar (if not exactly the same) to that of the preexcited beat could be an ectopy from the ablation catheter. It could also rarely be the result of Mahaim automaticity, which is often seen during ablation at the tricuspid annular site.

Following ablation, tachycardia was not inducible and preexcitation was not seen with right atrial pacing.

## Seeking answers

In our case, a final successful ablation was performed at a site other than the usual tricuspid annular site due to the lack of Mahaim potential recorded during tachycardia/right atrial pacing along the tricuspid annulus. In an effort to learn more from our peers, we recently sought to ask a panel of experts their opinions regarding the possible suitable options once no Mahaim potential can be documented along the tricuspid annulus. Their responses are available in the accompanying commentaries.
